# Memory Effect of Double Oxides Compared to Simple Ion Exchange for Controlled Fluoride Ion Capture and Release

**DOI:** 10.3390/ma18010162

**Published:** 2025-01-03

**Authors:** Asma Alazreg, Vladisav Tadić, Adela Egelja, Andrija Savić, Aleksandra Šaponjić, Marija M. Vuksanović, Radmila Jančić Heinemann

**Affiliations:** 1 Faculty of Technology and Metallurgy, University of Belgrade, 11000 Belgrade, Serbia; asmaalazreg@hotmail.com (A.A.); vladatadic98@gmail.com (V.T.); radica@tmf.bg.ac.rs (R.J.H.); 2 Department of Chemical Dynamics and Permanent Education, “VINČA” Institute of Nuclear Sciences—National Institute of the Republic of Serbia, University of Belgrade, 11000 Belgrade, Serbia; adela@vin.bg.ac.rs (A.E.); savic@vin.bg.ac.rs (A.S.); 3 Department of Materials, “VINČA” Institute of Nuclear Sciences—National Institute of the Republic of Serbia, University of Belgrade, 11000 Belgrade, Serbia; saponjicasandra75@gmail.com

**Keywords:** layered double hydroxide, memory effect, fluoride uptake and release

## Abstract

A layered double hydroxide (LDH) containing Mg and Al was synthesized from a nitrate solution using a coprecipitation method. The resulting material exhibited a homogeneous structure, which, upon calcination at 450 °C, was converted into a layered double oxide (LDO). When rehydrated in a fluoride-containing aqueous solution, the original hydroxide structure was successfully regenerated, demonstrating the LDH’s memory effect. During this transformation, fluoride anions from the solution were incorporated into the interlayer galleries to maintain electroneutrality, as confirmed by energy-dispersive X-ray spectroscopy (EDS) analysis. Separately, the process was tested in the presence of ethanol, which significantly enhanced the incorporation of fluoride ions into the interlayer spaces. The material’s potential for controlled fluoride release was evaluated by monitoring its release into demineralized water. For comparison, a simple ion-exchange process was carried out using the as-synthesized MgAl LDH. The memory effect mechanism displayed a notably higher fluoride incorporation capacity compared to the ion-exchange process. Among all the specimens, the sample reconstructed in the presence of ethanol exhibited the highest fluoride ion content. Fluoride release studies revealed a two-phase pattern: an initial rapid release within the first three hours, followed by a substantially slower release over time.

## 1. Introduction

Fluoride removal and release are key areas of study in environmental engineering, addressing the presence of fluoride in potable water [1,2]. These processes are also of interest in dentistry, where the controlled release of small fluoride concentrations can be beneficial, particularly when applied near dental tissues requiring mineral maintenance for optimal surface composition [3,4]. However, excessive fluoride intake can cause fluorosis, which adversely affects bone health and leads to overall toxicity [5]. Therefore, there is a continuous need to develop and optimize materials capable of effectively regulating these processes.

Layered double hydroxides (LDHs) are a class of materials capable of incorporating anions of varying sizes within their structure [6]. The general formula of an LDH can be written as [M^2+^_1−x_M^3+^_x_(OH)_2_]^x+^ [A^n−^]x/n·mH_2_O, where M^2+^ and M^3+^ are divalent and trivalent cations, A^n−^ is an n-valent anion incorporated in the interlayered space, and x usually has a value between 0.20 and 0.33 [7]. The structural similarity of LDHs to clay materials stems from their composition, which includes layers of trivalent oxides such as alumina combined with divalent metal oxides [8,9], depending on the synthesis method employed [10]. Due to their structure, they are suitable to be used in different environmental applications, where their behavior as semiconductors can be used to remove CO_2_ from atmosphere [11]. Alumina, a well-known adsorbent for various pollutants, exhibits enhanced potential when combined with other metal oxides [12]. LDHs are typically synthesized from precursors that are salts of divalent and trivalent cations. During synthesis, anions present in the solution are intercalated between the layers, though they are only weakly bound to the structure [13]. When these materials are exposed to environments containing different anions, ion exchange occurs, replacing the original anions with new ones [14]. For example, LDHs prepared from nitrate solutions retain nitrate ions between their layers, which can subsequently be exchanged for fluoride ions, resulting in the partial incorporation of fluoride into the structure. LDHs exhibit structural stability and hold promise for various practical applications. Upon calcination, LDHs are transformed into mixed oxides with a large specific surface area and significant pore volume [15]. This thermal treatment converts the layered double hydroxide structure into a mixture of oxides and spinel phases, which can be identified via XRD analysis. When these calcined materials are re-exposed to water, the original layered structure can be reconstituted—a phenomenon known as the “memory effect” [16,17]. The memory effect facilitates the incorporation of various anions into the structure during rehydration. Studies have shown that LDHs initially intercalated with organic anions are more likely to reconstruct their original structure upon rehydration. Additionally, materials containing carbonate anions readily reincorporate dissolved carbonate ions during the process [18]. This “memory effect” phenomenon is also observed in layered double oxides (LDOs) synthesized via sol–gel techniques [19].

Different methods for rehydrating MgAl layered double hydroxides (LDHs) were tested in combination with zeolite. The optimal rehydration of MgAl/LDHs was achieved at 300 °C in an inert atmosphere under a flow of He gas. The modified LDH structure, when combined with zeolite, demonstrated rehydration capabilities in an oxidative atmosphere, with heat treatment up to 500 °C [20]. The memory effect-based method for chloride ion removal was an economic and efficient technique for water treatment [21].

The combination of LDHs with zeolite offers potential for simultaneous anion and cation exchange, as demonstrated in previous research [22]. It is hypothesized that in water containing fluoride, this composite will retain fluoride ions, and its retention rate can be compared to that of traditional ion-exchange mechanisms.

An additional feature of this structure is its potential for controlled fluoride ion release, enabling precise dosing where needed. These hypotheses are tested using a system of MgAl LDHs and their corresponding oxides. The process is monitored by analyzing their structure and measuring ion concentrations in both the solid materials and the solutions they are in contact with.

## 2. Materials and Methods

### 2.1. Materials

The precursor materials, magnesium nitrate (Mg(NO_3_)_2_·6H_2_O) and aluminum nitrate (Al(NO_3_)_3_·9H_2_O) (Sigma Aldrich, St. Louis, MO, USA) were used for the preparation of a magnesium–aluminum layered double hydroxide (MgAl/LDH). Nitrate salts were selected due to their suitability for ion-exchange studies [23]. A sodium fluoride (Merck, Darmstadt, Germany) solution was then used to investigate the ion-exchange characteristics of the MgAl/LDH sample.

### 2.2. Preparation of Layered Structures

#### Coprecipitation Preparation of MgAl/LDH

**Figure 1:**
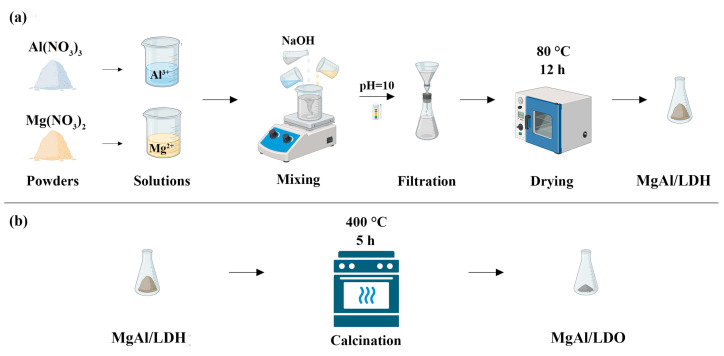
Schematic representation of (**a**) the synthesis of LDH via coprecipitation and (**b**) the calcination of LDH to obtain LDO.

The magnesium–aluminum layered double hydroxide (MgAl/LDH) was synthesized via the coprecipitation method, with a molar ratio of Mg/Al = 3:1. The precursor nitrate salts of the selected cations were dissolved in demineralized water and continuously stirred at room temperature. A 1.0 mol/L sodium hydroxide solution was gradually added to the mixture, maintaining a pH of 10 to initiate the precipitation process. The solution was left to age for 24 h to ensure complete precipitation. The resulting precipitate was then filtered, thoroughly washed with demineralized water until a neutral pH was achieved, and subsequently dried in an oven at 80 °C for 12 h (Figure 1a) [24,25,26].

Following synthesis, half of the MgAl/LDH was calcined at 400 °C for 5 h, resulting in the transformation of the LDH into a mixed metal oxide (MgAl/LDO) (Figure 1b) [27].

### 2.3. Ion Exchange for MgAl/LDH

Fluoride ions were used to investigate the ion-exchange characteristics of the layered double hydroxide (LDH) sample. A 0.5 g portion of the sample was added to 80 mL of a 0.149 mol/L sodium fluoride (NaF) solution and kept at room temperature for 24 h. The amount of fluoride ions captured during the LDH reconstruction process was quantified using EDS. Two different methods for fluoride uptake were tested: one using the NaF aqueous solution [28] and another using a mixture of the NaF solution with ethanol [29]. The rest of the procedure remained the same.

The reconstructed LDH material was then dried at 80 °C and tested for fluoride uptake through structural analysis. Fluoride release from the material was assessed by immersing the dried sample in demineralized water and monitoring the fluoride anion concentration in the solution using ion chromatography (IC).

### 2.4. Memory Effect Testing for MgAl/LDO

**Figure 2:**
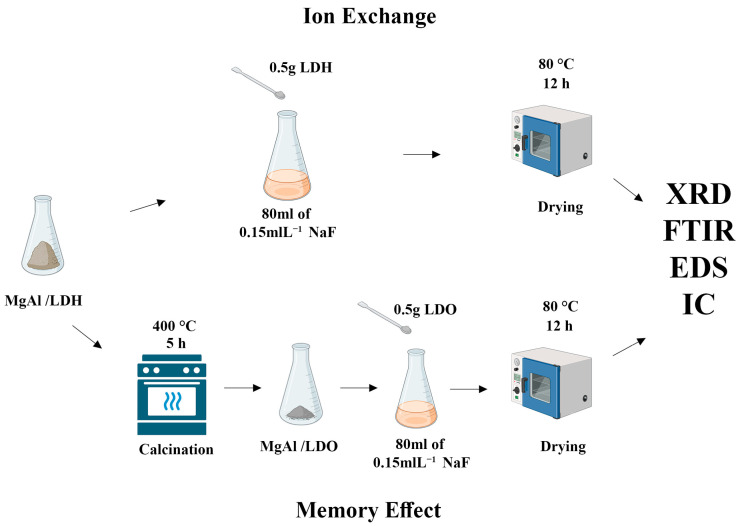
Schematic representation of ion exchange and the memory effect of the LDH.

The MgAl/LDH synthesized by the coprecipitation method was calcined at 400 °C for 5 h to obtain a layered double oxide (LDO) (Figure 2). The “memory effect” of LDHs, which allows the structure to return to its original layered form upon exposure to water, served as the basis for this experiment. Fluoride uptake was carried out in a 0.149 mol/L fluoride ion solution for 30 min [30]. The sample was then dried at 80 °C and tested to quantify fluoride uptake. The same procedure was repeated using a solution where half of the solvent was ethanol.

### 2.5. Characterization Methods

Morphological and elemental analyses of both MgAl/LDH and MgAl/LDO particles before and after adsorption were conducted using a 20 kV MIRA3 TESCAN (Oxford, UK) field-emission scanning electron microscope (FE-SEM) coupled with energy-dispersive X-ray spectroscopy (EDS). EDS analysis was performed using the INCAx-act LN2-free Analytical Silicon Drift Detector (Oxford Instruments, Oxford, UK) for characteristic X-ray detection, employing PentaFET^®^ Precision and Aztec 4.3 software (Oxford Instruments, Abingdon, UK) and connected to a TESCAN Mira3 XMU system (Oxford, UK)).

The crystal structures of the MgAl/LDH and MgAl/LDO particles were examined by X-ray diffraction (XRPD) using an Ultima IV Rigaku diffractometer (Tokyo, Japan) equipped with Bragg–Brentano geometry and CuKα radiation (λ = 1.5418 Å) in step-scan mode (range: 5–80° 2θ, step time: 0.50 s, step width: 0.02°).

The presence of chemical bonds was assessed using Fourier-transform infrared (FTIR) spectrometry. A Nicolet™ iS™ 10 FTIR spectrometer (Thermo Fisher Scientific, Waltham, MA, USA) equipped with Smart iTR™ attenuated total reflectance (ATR) sampling accessories was used. Spectra were recorded in the range of 4000–400 cm^−1^ with 20 scans and a resolution of 4 cm^−1^.

Fluoride concentrations in the solutions were analyzed using ion chromatography with a Dionex IonPac AS 14 (Orpington, Kent, UK) (4 × 250 mm) column and a suppressed conductivity detector.

## 3. Results and Discussion

**Figure 3:**
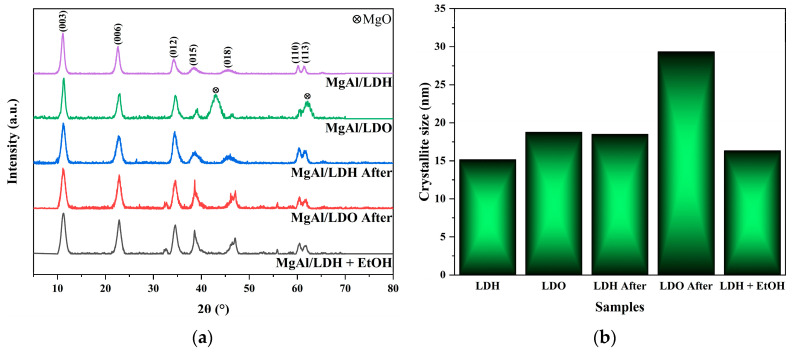
(**a**) XRD patterns of MgAl/LDH and MgAl/LDO samples and (**b**) corresponding crystallite sizes before and after fluoride treatment, calculated using the Scherrer equation.

The coprecipitation process is a well-established method for synthesizing layered hydroxide structures. These structures consist of alternating layers of aluminum and magnesium oxides, with intercalated anions ensuring overall electroneutrality. The crystalline structure can be elucidated using XRD analysis. Figure 3 displays the diffraction patterns for the as-synthesized MgAl/LDH samples, confirming the characteristic peaks of layered materials [31].

The calcined product is denoted as MgAl/LDO. After immersing the MgAl/LDH in a fluoride ion solution, the XRD peak positions remained unchanged; however, the peak intensities increased, suggesting enhanced crystallinity upon fluoride exposure. The characteristic peaks corresponding to the (003) and (006) planes of the LDH structure remained prominently visible.

For MgAl/LDO, the calcined form of MgAl/LDH, the XRD patterns reveal peaks at positions like those of MgAl/LDH. Additionally, two new peaks appear at approximately 43.2° and 62.4°, corresponding to the (200) and (220) planes of MgO (JCPDS card No. 77-2364) [32]. These findings indicate that during calcination, an oxide phase begins to develop from the layered structure, signifying a phase transformation from the original MgAl/LDH to an oxide-dominated structure. Furthermore, the (018) crystallographic plane of MgAl/LDH exhibits a noticeable shift in the MgAl/LDO material, further confirming the onset of a structural transformation during calcination.

The fluoride uptake was evaluated for the two materials, MgAl/LDH and MgAl/LDO, by monitoring crystallographic changes following their exposure to a NaF aqueous solution. After fluoride exposure, the peaks associated with the MgO phase in the MgAl/LDO material disappeared, and the diffraction pattern became identical to that of MgAl/LDH. This confirms the successful activation of the “memory effect”, wherein the MgAl/LDO structure reverts to its original LDH form upon fluoride uptake.

Fluoride uptake was also tested in a solution where half of the solvent was replaced with ethanol. Alcohols such as ethanol and methanol are hypothesized to influence the arrangement of the crystal structure in double-oxide systems. The XRD patterns of MgAl/LDO exposed to the mixed-solvent NaF solution revealed that the original MgAl/LDH structure was distinctly reconstructed.

For comparison, the same test was conducted with MgAl/LDH. The resulting structure exhibited peaks that were more pronounced than those in the original MgAl/LDH. This observation suggests that the presence of ethanol in the solution enhances the formation of a more well-defined crystalline structure compared to that in the MgAl/LDH exposed to an aqueous NaF solution for ion exchange.

The reconstruction of the MgAl/LDH structure from MgAl/LDO in the mixed-solvent NaF solution resulted in a highly defined crystalline structure with sharp characteristic peaks, as illustrated in Figure 3a.

The crystallite sizes of the samples were calculated using the Scherrer equation (Equation (1)), which was derived from the XRD data. This equation is widely employed to estimate the crystallite size in materials by analyzing the broadening of X-ray diffraction peaks.

(1) $$D=\frac{K\lambda }{\beta \cos\theta }$$

where D represents the average crystallite size, and *K* is a dimensionless shape factor commonly known as the Scherrer constant, with a typical value of approximately 0.9. *λ* is the wavelength of the X-rays used in the diffraction experiment, *β* is the full width at half of the maximum (FWHM) diffraction peak, and *θ* is the Bragg angle corresponding to the peak position.

The crystallite size serves as an indicator of the crystal structure’s definition. Among all the structures observed, the MgAl/LDH material initially exhibited the smallest crystallite size. The ion-exchange process, where nitrate ions in the interlayer spaces are replaced by fluoride ions, induced an increase in crystallite size. The calcined form of MgAl/LDH, denoted as MgAl/LDO, exhibited slightly larger crystallites than those in the as-synthesized MgAl/LDH. The exposure of MgAl/LDO to a fluoride aqueous solution led to the formation of larger crystallites compared to those in all previously observed structures. This phenomenon is attributed to the “memory effect”, which is believed to be driven by the recrystallization process [33]. According to this study, the first signs of recrystallization are observable within 30 min, which corresponds to the duration allotted to specimens during fluoride uptake. The crystallite size in specimens exposed to a mixed-solvent solution of water and ethanol also followed the same trend of crystallite size increase observed in the single-solvent NaF solution.

**Figure 4:**
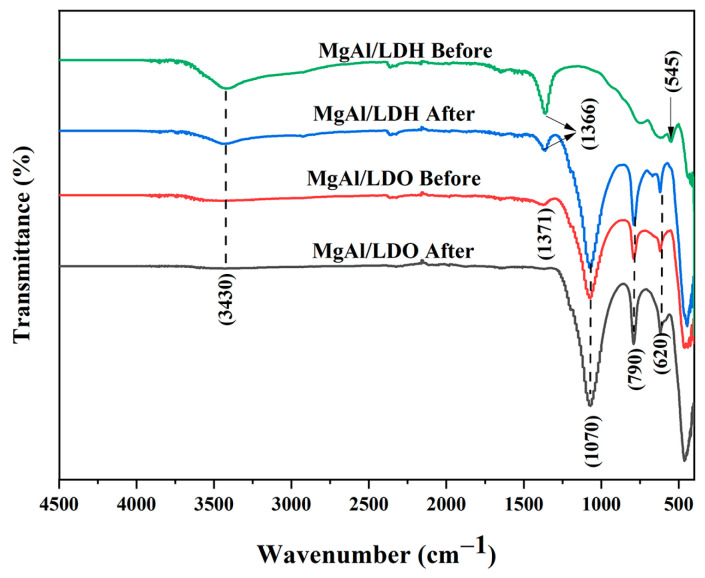
FTIR spectra of MgAl/LDH and MgAl/LDO particles with ethanol before and after fluoride treatment.

Figure 4 presents the FTIR spectra for both layered double hydroxide and oxide materials before and after fluoride treatment. The spectra reveal several characteristic peaks common to all samples. Notably, a broad peak around 3400 cm^−1^ is observed, which corresponds to O-H stretching vibrations and the stretching vibrations of interlayer water molecules [34]. Fluoride ions may cause modest shifts in the broad O-H stretching band about 3430 cm^−1^ in LDH samples prior to calcination. While the major attribution for this peak is water or hydroxyl groups, fluoride can interact with hydroxyl groups in the structure, perhaps moving this band somewhat. The FTIR spectrum does not exhibit a significant band about 430–500 cm^−1^, which is usually where the O-F stretching vibration for fluoride appears. This shows that fluoride is not present in the sample as a free ion but rather incorporated into the interlayer space of the LDH or LDO structure, where it may not create a noticeable FTIR characteristic peak. It may also be obscured by other prominent peaks or interactions within the spectrum. Fluoride ion intercalation into LDH is symbolized by the distinctive band at frequency 1385 cm^−1^ [35]. Visible bands around 630 cm^−1^ and 550 cm^−1^ correspond to Mg-OH and Al-OH translational vibrations, respectively [36]. These bands are characteristic of the metal hydroxide bonds within the LDH structure. For the MgAl/LDH material, no significant changes in the FTIR spectrum were observed before and after fluoride treatment, indicating that the structural integrity of the MgAl/LDH remained largely unaffected by the fluoride ions.

However, for the MgAl/LDO material, a decrease in the intensity of the O-H stretching peak at 3400 cm^−1^ is observed after calcination. This decrease is due to the removal of hydroxyl groups and interlayer water during the calcination process, which transforms the MgAl/LDH into its oxide form, MgAl/LDO.

**Figure 5:**
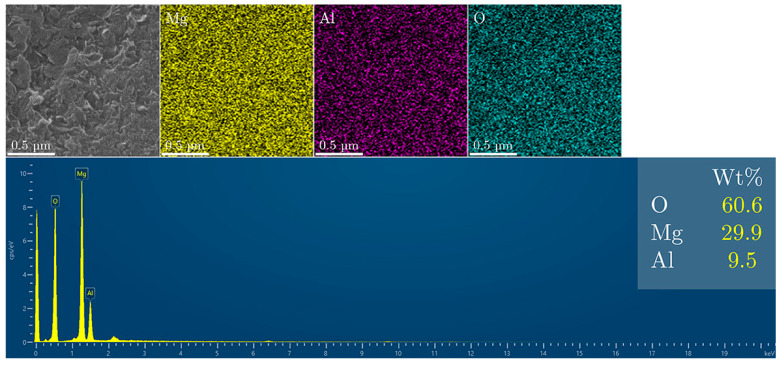
SEM and EDS analysis of the MgAl/LDH particles..

The as-prepared LDH particles were analyzed using scanning electron microscopy (SEM) to assess the morphology of the material and examine its elemental composition. The analysis revealed that the material primarily consisted of aluminum, magnesium, and the corresponding amounts of oxygen. No other elements were detected, which aligns with the synthesis method used (Figure 5). The SEM micrograph of the MgAl/LDH sample (Figure 5) shows a uniform, layered morphology, consistent with the typical structure of layered double hydroxides.

**Figure 6:**
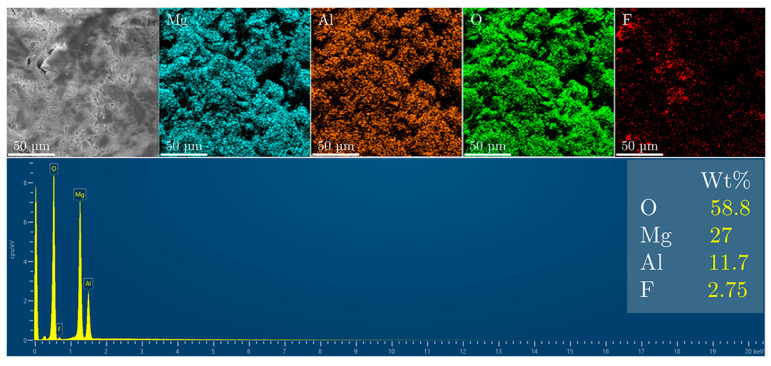
SEM and EDS analysis of the MgAl/LDH sample after fluoride treatment.

Fluoride uptake by the LDH structure was evaluated using SEM and energy-dispersive X-ray spectroscopy (EDS) after exposure to fluoride (Figure 6). The EDS spectrum of the sample indicates a dominant presence of oxygen (58.8%), along with significant amounts of magnesium (27%) and aluminum (11.7%). The fluoride content is relatively low, at 2.75%, which corresponds to the capacity for fluoride ion incorporation via the ion-exchange mechanism.

**Figure 7:**
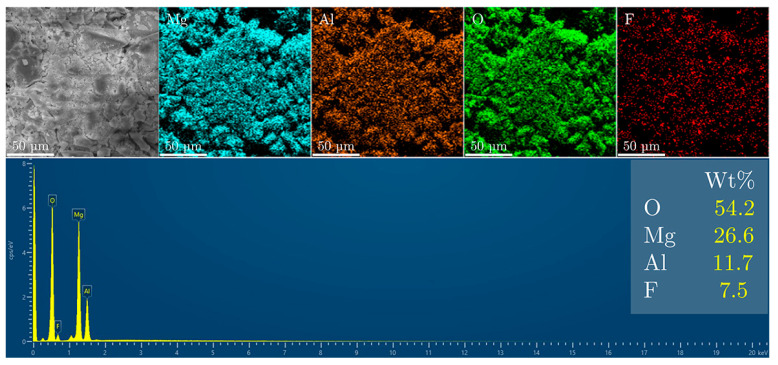
SEM and EDS analysis of the MgAl/LDO sample after fluoride treatment.

For the MgAl/LDH sample (Figure 7), the SEM micrograph reveals a surface morphology characterized by a mix of fine and coarse particles, reflecting the transformation of the MgAl/LDH structure after calcination. The observed texture is consistent with the formation of oxide phases and the disruption of the layered structure, which are typical during the transition from MgAl/LDH to MgAl/LDO. The EDS spectrum for this sample indicates the elemental composition as follows: oxygen (54.2%), magnesium (26.6%), aluminum (11.7%), and fluoride (7.5%).

The higher fluoride content in the MgAl/LDO sample compared to that in the MgAl/LDH suggests increased fluoride uptake. This is likely due to the more open structure and larger surface area of the MgAl/LDO material, which facilitates better interaction with fluoride ions. These findings indicate that the memory effect of MgAl/LDO is more efficient for fluoride uptake than the ion-exchange process of MgAl/LDH.

**Figure 8:**
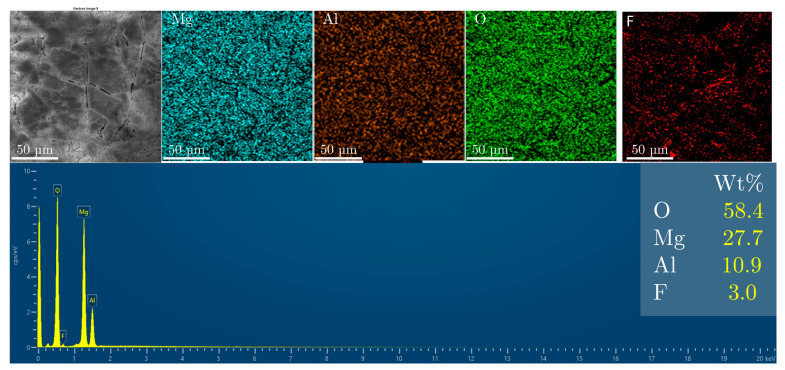
SEM and EDS analysis of the MgAl/LDH sample with ethanol after fluoride treatment.

For comparison, the MgAl/LDO sample was immersed in a mixed-solvent solution of water and ethanol containing fluoride, and it exhibited 10% more fluoride uptake than the original LDH sample. The presence of ethanol enhanced the ion-exchange mechanism, facilitating greater fluoride uptake. The results are shown in Figure 8.

**Figure 9:**
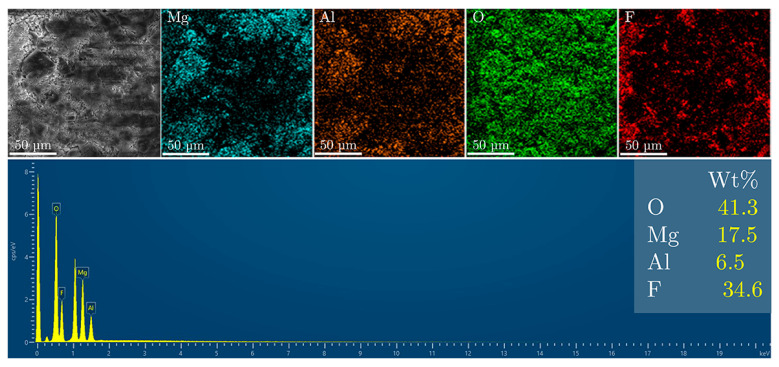
SEM and EDS analysis of the MgAl/LDO sample with ethanol after fluoride treatment.

The reconstruction of the LDO structure in the mixed ethanol–water NaF solution resulted in a higher level of fluoride uptake compared to that in both the LDO and LDH samples. This suggests that this method could be effective for charging the particles with a high fluoride content for controlled release in future applications. The morphology of the particles corresponds to that of the LDH structure reconstituted from the LDO in a water-only solution, as shown in the results presented in Figure 9.

### Fluoride Release

Considering that the memory effect of MgAl/LDO is more effective for fluoride uptake than the ion-exchange mechanism of MgAl/LDH and that the most efficient way to load fluoride ions into the structure is by immersion in a mixture of NaF, water, and ethanol, the obtained samples were immersed in demineralized water for 24 h to evaluate their fluoride release capacity. Following immersion, ion chromatography (IC) was employed to measure the concentrations of fluoride ions released from the MgAl/LDH and MgAl/LDO materials and compare them with those of samples immersed in the NaF–ethanol solution mixture.

**Figure 10:**
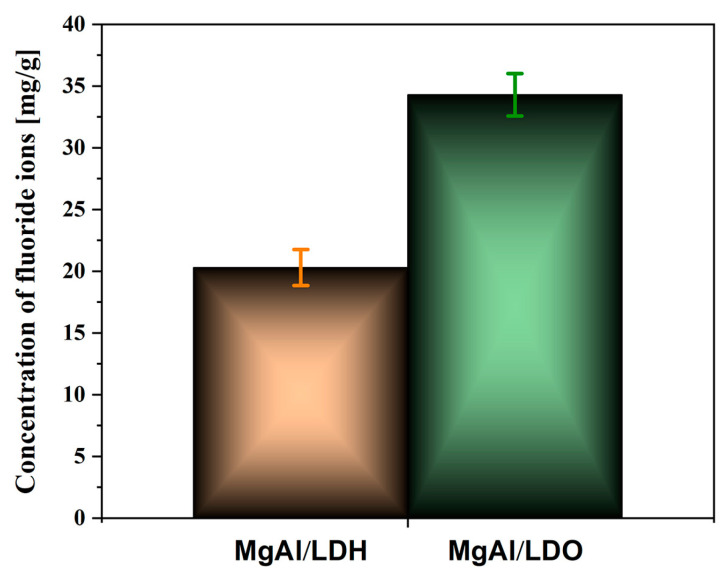
Concentration of fluoride ions for MgAl/LDH and MgAl/LDO particles.

Figure 10 presents the IC results, showing the fluoride ion concentration released from the MgAl/LDH and MgAl/LDO samples after 24 h of immersion in demineralized water. The MgAl/LDO material released a higher amount of fluoride ions than MgAl/LDH, which can be attributed to their distinct mechanisms.

For MgAl/LDH, fluoride ions were initially adsorbed via an ion-exchange process, where fluoride ions replaced nitrate (NO_3_^−^) ions in the interlayer spaces. Upon immersion in water, ion exchange took place, and the fluoride ions were released into the water.

In contrast, the MgAl/LDO material, after being calcined, underwent a disruption of its original layered structure, forming an oxide phase. This effect has been used in several industrial processes [37]. The reconstruction of the structure was possible due to recrystallization, which enhances the material reactivity and possible activation of active sites [38]. Following exposure to a fluoride solution, the memory effect allowed the MgAl/LDO to revert to its layered structure, where it was able to adsorb fluoride ions. The reversion of the structure occurred at the same time as the ion adsorption, as the oxides were in their metastable form and had a large specific surface area, so the incorporation of anions in the vicinity was easier compared to the process in the layered form of the material.

**Figure 11:**
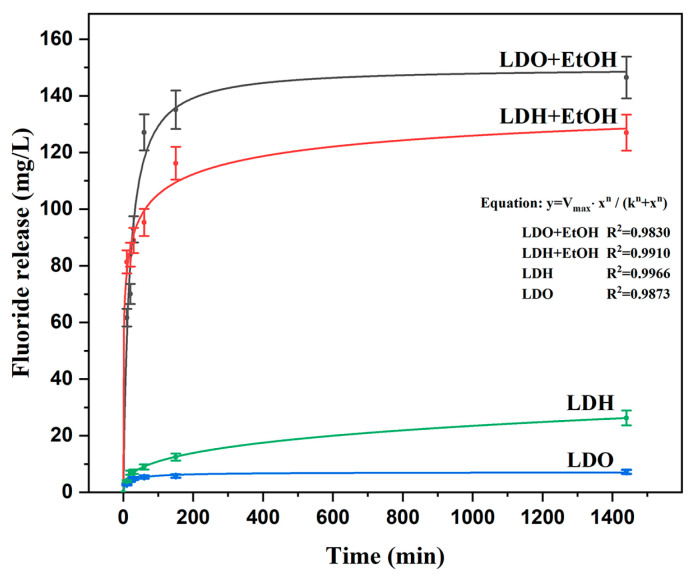
Fluoride release for MgAl/LDH and MgAl/LDO particles and samples with ethanol.

The MgAl/LDO material released a higher fluoride ion content compared to MgAl/LDH, likely due to the calcination process, which increased the number of available active sites for ion exchange. The higher fluoride uptake observed in the solid samples treated with the water–ethanol solution was further tested in a fluoride release experiment, and the release profiles were compared to those from the LDH ion-exchange and LDO memory-effect fluoride capture (Figure 11). Fluoride release was rapid during the first 3 h, after which it plateaued, with little change in the concentration of fluoride ions in the solution. The fluoride release from specimens treated with the ethanol solution was significantly more intense, mirroring the fluoride uptake observed previously.

It was previously shown that alcohols play an important role in the synthesis of CoNi LDH structures, enabling control of the morphology and activity of the material [39]. Ethanol and methanol play crucial roles in the transformation of layered double hydroxides (LDHs) to layered double oxides (LDOs) and vice versa, particularly in influencing the memory effect. This phenomenon refers to the ability of calcined LDHs (LDOs) to rehydrate and reconstruct their original layered structure when exposed to water or other solvents. Alcohols such as ethanol and methanol can act as co-solvents, enhancing the dispersion of materials during rehydration. They facilitate the interaction between water molecules and the oxide matrix, promoting the formation of hydroxyl groups essential for the reconstruction of the LDH structure. Alcohols can also influence the surface properties and functional group interactions within the interlayer spaces, affecting phase transformation kinetics and improving the structural uniformity of the regenerated LDH. In catalytic applications, the presence of methanol or ethanol during the LDH-LDO transformation can alter the material’s porosity, surface area, and active site distribution, ultimately impacting its performance in processes such as the hydrogenation or electrooxidation of methanol [40].

A review of the literature reveals several materials proposed for fluoride capture. One notable example is the 2,5-thiophenedicarboxylate (H_2_TDC) ligand, which has been used to prepare a series of TDC-MOFs by varying the central metal ions (e.g., Al^3+^, Ce^4+^, and Zr^4+^), as discussed in [41]. Although this material demonstrates high sensitivity for fluoride capture, it is relatively complex to synthesize and more sensitive compared to simpler alternatives like LDH (layered double hydroxide) materials. Other studies focus on hybrid materials combining LDH with additional sorbents. For instance, a Co/Fe-LDH@Avocado kernel seed biochar@Carboxymethylcellulose nanobiosorbent has been explored, where fluoride sorption primarily occurs due to the LDH component, while the cellulose serves as an eco-friendly structural support [42]. Another approach involves using natural hydrogels as matrices for mineral-based absorbents targeting fluoride and phosphate anions [43]. Natural non-metallic minerals, characterized by their abundant reserves, low cost, and environmental compatibility, are increasingly recognized as promising candidates for adsorption applications. Their unique surface charges, active adsorption sites, and surface activity make them cost-effective, efficient, and safe options for capturing various pollutants. This paper reviews recent progress in the use of natural non-metallic minerals (e.g., montmorillonite, kaolinite, attapulgite, bentonite, and zeolite) and their derivatives for the adsorption and removal of fluoride and phosphate pollutants over the past decades [44].

## 4. Conclusions

MgAl/LDH and MgAl/LDO were synthesized via the coprecipitation process, followed by calcination for the MgAl/LDO. The materials’ capacities to take up fluoride ions were compared using elemental analysis, which demonstrated that the LDO exhibited a greater affinity for fluoride incorporation, resulting in higher fluoride concentrations in the structure, as confirmed by EDS analysis. The process conducted in a mixed-solvent solution of fluoride yielded improved results in terms of fluoride uptake, with higher concentrations observed in the solid material. Fluoride anion release into demineralized water was notably higher from the LDO structure, with increased concentrations measured after 24 h. Additionally, the crystal structure of the LDO was re-established after contact with the fluoride solution, and the crystallite size increased, confirming both crystal stabilization and enhanced fluoride uptake.

## Data Availability

The data presented in this study are available on request from the corresponding author or co-authors. The data are not publicly available because the work is related to dr. thesis of the first author.
